# Supplementary material for: Influence of Viral Re-Infection on Head Kidney Transcriptome of Nervous Necrosis Virus-Resistant and -Susceptible European Sea Bass (Dicentrarchus labrax, L.)
Source: Viruses. 2025 Feb 7;17(2):230. doi: 10.3390/v17020230 (PMC11860166; doi:10.3390/v17020230)
Supplement: Supplementary file 1 [file viruses-17-00230-s001.zip › viruses-3422163-supplementary.pdf]

viruses

MDPI

Article

Influence of Viral Re-Infection on Head Kidney Transcriptome of Nervous Necrosis Virus Resistant and Susceptible European Sea Bass (*Dicentrarchus labrax, L.*)

Dimitra K. Toubanaki [1,*], Odysseas-Panagiotis Tzortzatos [1], Antonia Efstathiou [1], Vasileios Bakopoulos[2] and Evdokia Karagouni[1,*]

[1] Immunology of Infection Group, Department of Microbiology, Hellenic Pasteur Institute, 11521 Athens, Greece; dtouban@pasteur.gr (D.K.T.); toniaef@pasteur.gr (A.E.); ptzortzatos@pasteur.gr (O.P.T).
[2] Department of Marine Sciences, School of The Environment, University of The Aegean, University Hill, Lesvos, Mytilene, 81100, Greece; v.bakopoulos@aegean.gr (V.B.)
* Correspondence: dtouban@pasteur.gr (D.K.T.) +30-2106478828; ekaragouni@pasteur.gr (E.K.), +30-2106478826.

Supplementary material

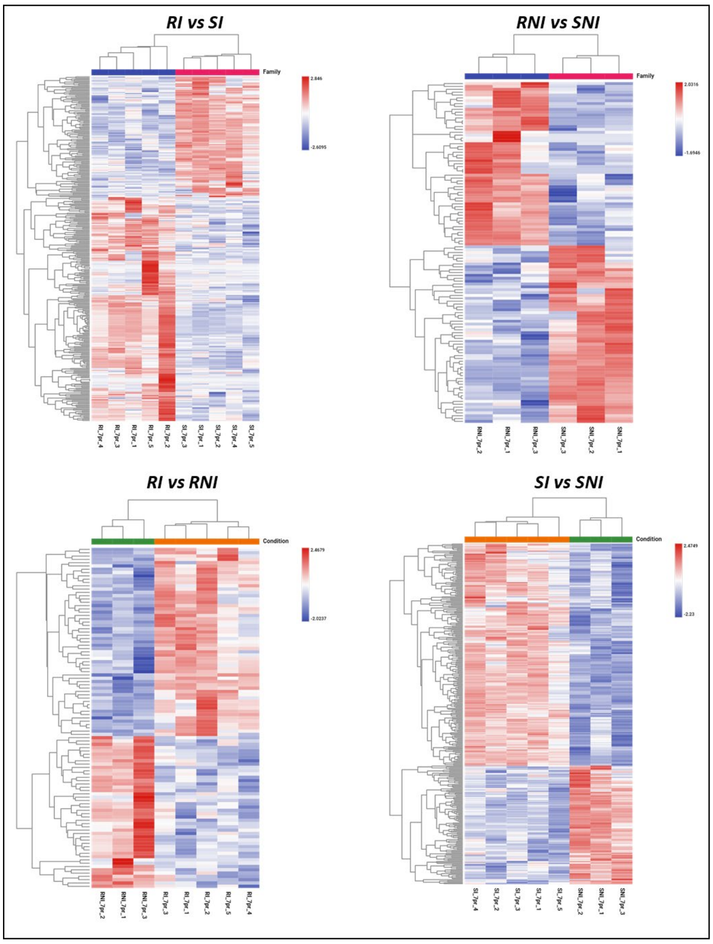

Figure S1. Cluster analysis of DEGs. Each column in the graph represents a sample, each row represents a gene, and the expression of genes in different samples is represented by different colors, with redder colors indicating higher expression and bluer colors indicating lower expression. Heatmap of the genes showed more than a 1.5-fold difference between the resistant (R: blue bar) and

susceptible (S: magenta bar) families or between the NNV infected (I: orange bar) and non-infected (NI: green bar) experimental groups. Red and blue colors indicate up and downregulation in Log2 cpm values, respectively. Each column in the graph represents a sample, each row rep-resents a gene, and the expression of genes in different samples is represented by different colors, with redder colors indicating higher expression and bluer colors indicating lower expression.

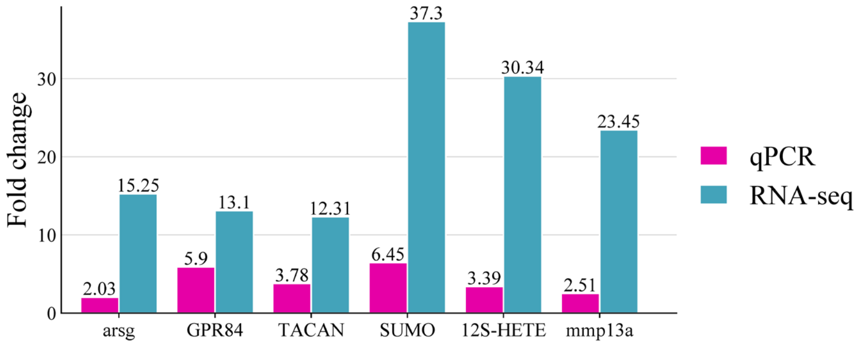

Figure S2. Validation of the RNA-Seq results by qPCR. Arsg: arylsulfatase g, GPR84: g-protein coupled receptor 84, TACAN: transmembrane protein 120a; SUMO: zinc finger bed domain-containing protein 1, 12S-HETE: hydroxycarboxylic acid receptor 2-like, mmp13: collagenase 3-like.

Table S1. Primers and probes used in the present work.

Gene	Primer sequence (5′ - 3′)	Accession number	Product size (bp)	Reference
NNV load quantification				
**Primers**	oPVP154: TCCAAGCCGGTCCTAGTCAA	N/A	168/171	[27]
	oPVP155: CACGAACGTKCGCATCTCGT			
**Taqman probe**	tqPVP16: Cy5-CGATCGATCAGCACCTSGTCBHQ2	N/A	N/A	[27]
qPCR validation				
**arsg**	Up: TGATGTGTGGTCTGCTTCTC	XM_051417193.1	107	Present work
	Dp: CCAGCCTATATCATCTGCCAATA			
**GPR84**	Up: CCCATCTCAGTTGACTCCTATCT	XM_051415820.1	115	Present work
	Dp: GGCAGAGGGTGATAATGGAAAC			
**TACAN**	Up: TGTGGTCTCTGCTTCCTTATTC	XM_051398431.1	93	Present work
	Dp: TGGTTTCTGATTCCTGCTCTT			
**SUMO**	Up: CAGCCAAGGTCTTGAGGAAG	XM_051406441.1	116	Present work
	Dp: CAGCCCCTGAGGTGTATGTT			
**12S-HETE**	Up: AGAAGACAGTGGGAGAGAAGA	XM_051409988.1	86	Present work
	Dp: CAGGAAGCAGATGGAGAAGAC			
**mmp13a**	Up: CGAGGATGAGACCTTCACTTTC	XM_051401301.1	118	Present work
	Dp: CATGAGAGCACCAGGATCATTAG			
Reference				
**EF-1a**	Up: CTCTTGATGCCATCCTGCCA	AJ866727.1	94	Present work
	Dp: ACAGTTCCAATACCGCCGAT			

* N/A: Non applicable.

GO Term	GO Name	GO Category	p-value	Adj. p-value (FDR)
GO:0005829	cytosol	CC	4.94E-29	1.26E-26
GO:0023052	signaling	BP	2.34E-27	5.96E-25
GO:0005886	plasma membrane	CC	4.25E-27	1.08E-24
GO:0005739	mitochondrion	CC	1.51E-20	3.85E-18
GO:0016787	hydrolase activity	MF	1.51E-20	3.85E-18
GO:0002376	**immune system process**	BP	7.41E-20	1.89E-17
GO:0048856	anatomical structure development	BP	5.44E-17	1.39E-14
GO:0016192	**vesicle-mediated transport**	BP	7.91E-16	2.02E-13
GO:0065003	protein-containing complex assembly	BP	1.67E-13	4.25E-11
GO:0031410	**cytoplasmic vesicle**	CC	9.11E-13	2.32E-10
GO:0005615	extracellular space	CC	5.87E-12	1.50E-09
GO:0012501	programmed cell death	BP	3.28E-11	8.36E-09
GO:0016491	oxidoreductase activity	MF	7.35E-11	1.87E-08
GO:0005783	endoplasmic reticulum	CC	1.47E-10	3.74E-08
GO:0006629	lipid metabolic process	BP	2.68E-10	6.84E-08
GO:0005198	structural molecule activity	MF	8.52E-10	2.17E-07
GO:0007005	mitochondrion organization	BP	8.52E-10	2.17E-07
GO:0030163	protein catabolic process	BP	8.52E-10	2.17E-07
GO:0030154	cell differentiation	BP	1.15E-09	2.94E-07
GO:0098542	defense response to other organism	BP	2.55E-09	6.50E-07
GO:0140096	catalytic activity, acting on a protein	MF	1.18E-08	3.02E-06
GO:0005975	carbohydrate metabolic process	BP	4.86E-08	1.24E-05
GO:0006520	amino acid metabolic process	BP	4.86E-08	1.24E-05
GO:0016740	transferase activity	MF	5.53E-08	1.41E-05
GO:0060089	molecular transducer activity	MF	1.19E-07	3.04E-05
GO:0006091	generation of precursor metabolites and energy	BP	1.94E-07	4.94E-05
GO:0005654	nucleoplasm	CC	1.94E-07	4.94E-05
GO:0055086	nucleobase-containing small molecule metabolic process	BP	1.94E-07	4.94E-05
GO:0005764	lysosome	CC	4.83E-07	1.23E-04
GO:1901135	carbohydrate derivative metabolic process	BP	4.83E-07	1.23E-04
GO:0003677	DNA binding	MF	4.83E-07	1.23E-04
GO:0022414	reproductive process	BP	1.68E-06	4.29E-04
GO:0098772	molecular function regulator activity	MF	2.68E-06	6.84E-04
GO:0048870	cell motility	BP	5.72E-06	1.46E-03
GO:0006325	chromatin organization	BP	1.35E-05	3.46E-03
GO:0006913	nucleocytoplasmic transport	BP	1.35E-05	3.46E-03
GO:0006914	autophagy	BP	1.35E-05	3.46E-03
GO:0006259	DNA metabolic process	BP	4.06E-05	1.03E-02
GO:0008092	cytoskeletal protein binding	MF	4.06E-05	1.03E-02
GO:0005856	cytoskeleton	CC	8.09E-05	2.06E-02
GO:0006886	intracellular protein transport	BP	8.09E-05	2.06E-02
GO:0061024	membrane organization	BP	1.35E-04	3.43E-02
GO:0006355	regulation of DNA-templated transcription	BP	1.35E-04	3.43E-02

Table S2. The Gene Ontology (GO) enriched terms in RI vs. RNI groups up-regulated DEGs. BP: Biological process, MF: molecular function, CC: cellular component.

GO Term	GO Name	GO Category	p-value	Adj. p-value (FDR)

GO:0005886	plasma membrane	CC	1.11E-36	2.84E-34
GO:0023052	signaling	BP	3.12E-34	7.96E-32
GO:0048856	anatomical structure development	BP	4.19E-31	1.07E-28
GO:0002376	**immune system process**	BP	1.66E-23	4.23E-21
GO:0060089	molecular transducer activity	MF	6.01E-21	1.53E-18
GO:0030154	cell differentiation	BP	1.68E-20	4.28E-18
GO:0005615	extracellular space	CC	1.01E-17	2.59E-15
GO:0055085	transmembrane transport	BP	4.82E-17	1.23E-14
GO:0048870	cell motility	BP	1.60E-16	4.09E-14
GO:0016740	transferase activity	MF	4.40E-16	1.12E-13
GO:0005215	transporter activity	MF	5.16E-16	1.32E-13
GO:0007155	cell adhesion	BP	1.44E-14	3.67E-12
GO:0006629	lipid metabolic process	BP	2.36E-13	6.01E-11
GO:0005783	endoplasmic reticulum	CC	4.81E-11	1.23E-08
GO:0005768	endosome	CC	3.52E-10	8.98E-08
GO:0005829	cytosol	CC	1.61E-09	4.10E-07
GO:0022414	reproductive process	BP	2.45E-09	6.26E-07
GO:0140096	catalytic activity, acting on a protein	MF	4.90E-09	1.25E-06
GO:0016491	oxidoreductase activity	MF	8.80E-09	2.24E-06
GO:0005634	nucleus	CC	2.29E-08	5.85E-06
GO:0005525	GTP binding	MF	2.52E-08	6.42E-06
GO:0140097	catalytic activity, acting on DNA	MF	2.52E-08	6.42E-06
GO:0048018	receptor ligand activity	MF	1.01E-07	2.56E-05
GO:0003013	circulatory system process	BP	1.01E-07	2.56E-05
GO:0050877	**nervous system process**	BP	1.01E-07	2.56E-05
GO:0030198	extracellular matrix organization	BP	1.01E-07	2.56E-05
GO:0061024	membrane organization	BP	2.51E-07	6.40E-05
GO:0006355	regulation of DNA-templated transcription	BP	2.51E-07	6.40E-05
GO:0012501	programmed cell death	BP	1.40E-06	3.56E-04
GO:0065003	protein-containing complex assembly	BP	2.09E-06	5.33E-04
GO:0016192	**vesicle-mediated transport**	BP	2.98E-06	7.60E-04
GO:0005739	mitochondrion	CC	5.44E-06	1.39E-03
GO:0016787	hydrolase activity	MF	5.44E-06	1.39E-03
GO:0003012	muscle system process	BP	8.79E-06	2.24E-03
GO:0050886	endocrine process	BP	8.79E-06	2.24E-03
GO:0034330	cell junction organization	BP	8.79E-06	2.24E-03
GO:0006954	inflammatory response	BP	2.63E-05	6.71E-03
GO:0005794	Golgi apparatus	CC	2.63E-05	6.71E-03
GO:0006766	vitamin metabolic process	BP	2.63E-05	6.71E-03
GO:0031012	extracellular matrix	CC	2.63E-05	6.71E-03
GO:0008289	lipid binding	MF	2.63E-05	6.71E-03
GO:0005856	cytoskeleton	CC	5.25E-05	1.34E-02
GO:0006886	intracellular protein transport	BP	5.25E-05	1.34E-02
GO:0005764	lysosome	CC	8.74E-05	2.23E-02
GO:1901135	carbohydrate derivative metabolic process	BP	8.74E-05	2.23E-02
GO:0003677	DNA binding	MF	8.74E-05	2.23E-02
GO:0098542	**defense response to other organism**	BP	1.31E-04	3.34E-02

Table S3. The Gene Ontology (GO) enriched terms in RI vs. RNI groups down-regulated DEGs. BP: Biological process, MF: molecular function, CC: cellular component.

Database	Enriched pathway	Pathway top category	Pathway category
KEGG	Chemical carcinogenesis - reactive oxygen species	Human Diseases	Cancer: overview
	Central carbon metabolism in cancer		
	Non-small cell lung cancer		Cancer: specific types
	Thyroid cancer		
	Diabetic cardiomyopathy		Cardiovascular disease
	Lipid and atherosclerosis		
	Viral myocarditis		
	Non-alcoholic fatty liver disease		Endocrine and metabolic disease
	Type I diabetes mellitus		
	Graft-versus-host disease		Immune disease
	Allograft rejection		
	Autoimmune thyroid disease		
	Legionellosis		Infectious disease: bacterial
	Salmonella infection		
	Shigellosis		
	Pathogenic Escherichia coli infection		
	Yersinia infection		
	Pertussis		
	Measles		Infectious disease: viral
	Hepatitis B		
	Human immunodeficiency virus 1 infection		
	Epstein-Barr virus infection		
	Coronavirus disease - COVID-19		
	Herpes simplex virus 1 infection		
	Human cytomegalovirus infection		
	Influenza A		
	Prion disease		Neurodegenerative disease
	Parkinson disease		
	Alzheimer disease		
	Amyotrophic lateral sclerosis		
	Pathways of neurodegeneration - multiple diseases		
	Huntington disease		
	Spinocerebellar ataxia		
	Alcoholism		Substance dependence
	ATP-dependent chromatin remodeling	Genetic Information Processing	Chromosome
	Protein processing in endoplasmic reticulum		Folding, sorting and degradation
	Proteasome		
	RNA degradation		
	Ubiquitin mediated proteolysis		
	Fanconi anemia pathway		Replication and repair
	DNA replication		
	Nucleotide excision repair		
	Spliceosome		Transcription
	Basal transcription factors		
	RNA polymerase		
	Ribosome		Translation
	Nucleocytoplasmic transport		
	Aminoacyl-tRNA biosynthesis		

	mRNA surveillance pathway		
	Glycine, serine and threonine metabolism	Metabolism	Amino acid metabolism
	Arginine and proline metabolism		
	Valine, leucine and isoleucine degradation		
	Cysteine and methionine metabolism		
	Citrate cycle (TCA cycle)		Carbohydrate metabolism
	Glycolysis / Gluconeogenesis		
	Fructose and mannose metabolism		
	Pyruvate metabolism		
	Starch and sucrose metabolism		
	Oxidative phosphorylation		Energy metabolism
	Methane metabolism		
	Fatty acid degradation		Lipid metabolism
	Drug metabolism - cytochrome P450		Xenobiotics biodegradation and metabolism
	Biosynthesis of cofactors		
	Longevity regulating pathway - worm	Organismal Systems	Aging
	Cardiac muscle contraction		Circulatory system
	Axon guidance		Development and regeneration
	Cholesterol metabolism		Digestive system
	PPAR signaling pathway		Endocrine system
	Progesterone-mediated oocyte maturation		
	Estrogen signaling pathway		
	Thermogenesis		Environmental adaptation
	Circadian rhythm		
	Neutrophil extracellular trap formation		Immune system
	IL-17 signaling pathway		
	Antigen processing and presentation		
	C-type lectin receptor signaling pathway		
	Cytosolic DNA-sensing pathway		
	NOD-like receptor signaling pathway		
	Cell cycle	Cellular Processes	Cell growth and death
	Cell cycle - yeast		
	Meiosis - yeast		
	p53 signaling pathway		
	Apoptosis		
	Oocyte meiosis		
	Apoptosis - multiple species		
	Necroptosis		
	Cellular senescence		
	Motor proteins		Cell motility
	ABC transporters	Environmental Information Processing;	Membrane transport
	HIF-1 signaling pathway	Environmental Information Processing;	Signal transduction
	Phospholipase D signaling pathway		
	Apelin signaling pathway		
	Rap1 signaling pathway		
	Ras signaling pathway		
	Cytokine-cytokine receptor interaction		Signaling molecules and interaction
	Viral protein interaction with cytokine and cytokine receptor		

	Cell adhesion molecules		
Reactome	RND2 GTPase cycle	Signal Transduction	
	RAC2 GTPase cycle		
	RHOG GTPase cycle		
	RAC3 GTPase cycle		
	Signaling by Rho GTPases, Miro GTPases and RHOBTB3		
	Signaling by Rho GTPases		
	RHO GTPase cycle		
	Signaling by Non-Receptor Tyrosine Kinases		
	Signaling by PTK6		
	PTK6 Regulates RHO GTPases, RAS GTPase and MAP kinases		
	Apoptotic cleavage of cellular proteins	Programmed Cell Death	
	Apoptotic cleavage of cell adhesion proteins		
	Apoptotic execution phase		
	Apoptosis		
	Rap1 signalling	Immune system	
	Antigen processing: Ubiquitination & Proteasome degradation		
	Class I MHC mediated antigen processing & presentation		
	Formation of the cornified envelope	Developmental Biology	
	Platelet activation, signaling and aggregation	Hemostasis	

Table S4. Enriched pathway categories on KEGG and Reactome databases for RI vs. RNI groups.

GO Term	GO Name	GO Category	p-value	Adj. p-value (FDR)
GO:0023052	signaling	BP	2.42E-24	4.01E-20
GO:0006355	regulation of DNA-templated transcription	BP	2.71E-10	4.49E-06
GO:0140110	transcription regulator activity	MF	5.91E-10	9.79E-06
GO:0003677	DNA binding	MF	1.10E-09	1.82E-05
GO:0005525	GTP binding	MF	1.61E-09	2.68E-05
GO:0060089	molecular transducer activity	MF	1.84E-09	3.05E-05
GO:0048870	cell motility	BP	6.51E-09	1.08E-04
GO:0043231	intracellular membrane-bounded organelle	CC	1.08E-08	1.79E-04
GO:0016787	hydrolase activity	MF	2.10E-07	3.49E-03
GO:0048856	anatomical structure development	BP	2.20E-07	3.64E-03
GO:0003924	GTPase activity	MF	2.38E-07	3.94E-03
GO:0002376	**immune system process**	BP	2.57E-07	4.26E-03
GO:0098772	molecular function regulator activity	MF	5.43E-07	9.00E-03
GO:0005615	extracellular space	CC	6.44E-07	1.07E-02
GO:0030154	cell differentiation	BP	2.37E-06	3.93E-02
GO:0048018	receptor ligand activity	MF	2.40E-06	3.98E-02

Table S5. The Gene Ontology (GO) enriched terms in SI vs. SNI groups up-regulated DEGs. BP: Biological process, MF: molecular function, CC: cellular component.

GO Term	GO Name	GO Category	p-value	Adj. p-value (FDR)
GO:0005615	extracellular space	CC	7.47E-11	1.24E-06
GO:0009987	cellular process	BP	6.03E-10	1.00E-05

GO:0005886	plasma membrane	CC	8.82E-09	1.46E-04
GO:0005215	transporter activity	MF	2.97E-06	4.92E-02

Table S6. The Gene Ontology (GO) enriched terms in SI vs. SNI groups down-regulated DEGs. BP: Biological process, MF: molecular function, CC: cellular component.

Database	Enriched pathway	Pathway top category	Pathway category
KEGG	Lipid and atherosclerosis	Human Diseases	Cardiovascular disease
	Type I diabetes mellitus		Endocrine and metabolic disease
	Graft-versus-host disease		Immune disease
	Autoimmune thyroid disease		
	Allograft rejection		
	Systemic lupus erythematosus		
	Salmonella infection		Infectious disease: bacterial
	Pathogenic Escherichia coli infection		
	Pertussis		
	Yersinia infection		
	Shigellosis		
	Influenza A		Infectious disease: viral
	Coronavirus disease - COVID-19		
	Proteasome	Genetic Information Processing	Folding, sorting and degradation
	DNA replication		Replication and repair
	Nucleotide excision repair		
	RNA polymerase		Transcription
	Starch and sucrose metabolism	Metabolism	Carbohydrate metabolism
	Oxidative phosphorylation		Energy metabolism
	Nicotinate and nicotinamide metabolism		Metabolism of cofactors and vitamins
	Biosynthesis of cofactors		
	Longevity regulating pathway - worm	Organismal Systems	Aging
	GnRH signaling pathway		Endocrine system
	Prolactin signaling pathway		
	Estrogen signaling pathway		
	C-type lectin receptor signaling pathway		Immune system
	Cytosolic DNA-sensing pathway		
	NOD-like receptor signaling pathway		
	T cell receptor signaling pathway		
	IL-17 signaling pathway		
	Necroptosis (ko04217)	Cellular Processes	Cell growth and death
	Cell cycle (ko04110)		
	Cell cycle - yeast (ko04111)		
	Meiosis - yeast (ko04113)		
	Regulation of actin cytoskeleton (ko04810)		Cell motility
	Tight junction (ko04530)		Cellular community - eukaryotes
Reactome	Cytokine Signaling in Immune system	Immune system	
	Signaling by Interleukins		
	MyD88 dependent cascade initiated on endosome		
	TRAF6 mediated induction of NFkB and MAP kinases upon TLR7/8 or 9 activation		
	Toll-like Receptor Cascades		
	TRIF(TICAM1)-mediated TLR4 signaling		
	Toll Like Receptor TLR1:TLR2 Cascade		

	Toll Like Receptor 3 (TLR3) Cascade		
	MyD88:MAL(TIRAP) cascade initiated on plasma membrane		
	MyD88-independent TLR4 cascade		
	MyD88 cascade initiated on plasma membrane		
	Toll Like Receptor 4 (TLR4) Cascade		
	Toll Like Receptor 7/8 (TLR7/8) Cascade		
	Toll Like Receptor 5 (TLR5) Cascade		
	Toll Like Receptor TLR6:TLR2 Cascade		
	Toll Like Receptor 2 (TLR2) Cascade		
	Toll Like Receptor 9 (TLR9) Cascade		
	Toll Like Receptor 10 (TLR10) Cascade		
	Interleukin-1 family signaling		
	Regulation of NF-kappa B signaling		
	Interleukin-1 signaling		
	TAK1-dependent IKK and NF-kappa-B activation		
	Innate Immune System		
	Glutathione conjugation	Metabolism	
	Glutathione synthesis and recycling		
	Biological oxidations		
	Phase II - Conjugation of compounds		
	Aflatoxin activation and detoxification		
	NRIF signals cell death from the nucleus	Signal Transduction	Signal Transduction (Gallus gallus)
	NRIF signals cell death from the nucleus		Signal Transduction (Rattus norvegicus)
	NRIF signals cell death from the nucleus		Signal Transduction (Canis familiaris)
	NRIF signals cell death from the nucleus		Signal Transduction (Mus musculus)
	Post-translational protein modification	Metabolism of proteins	
	Paracetamol ADME	Drug ADME	
	Generic Transcription Pathway	Gene expression	Transcription

Table S7. Enriched pathway categories on KEGG and Reactome databases for SI vs. SNI groups.

References

27. Baud, M.; Cabon, J.; Salomoni, A.; Toffan, A.; Panzarin, V.; Bigarré L. First generic one step real-time Taqman RT-PCR targeting the RNA1 of betanodaviruses. *J Virol Methods*. **2015**, *211*, 1-7. doi: 10.1016/j.jviromet.2014.09.016.

Disclaimer/Publisher's Note: The statements, opinions and data contained in all publications are solely those of the individual author(s) and contributor(s) and not of MDPI and/or the editor(s). MDPI and/or the editor(s) disclaim responsibility for any injury to people or property resulting from any ideas, methods, instructions or products referred to in the content.
